# Elevated CO_2_ influences microbial carbon and nitrogen cycling

**DOI:** 10.1186/1471-2180-13-124

**Published:** 2013-05-29

**Authors:** Meiying Xu, Zhili He, Ye Deng, Liyou Wu, Joy D van Nostrand, Sarah E Hobbie, Peter B Reich, Jizhong Zhou

**Affiliations:** 1 State Key Laboratory of Applied Microbiology (Ministry—Guangdong Province Jointly Breeding Base), South China, Guangdong Institute of Microbiology, Guangzhou, China; 2 Institute for Environmental Genomics and Department of Botany and Microbiology, University of Oklahoma, Norman, USA; 3 Department of Ecology, Evolution, and Behavior, University of Minnesota, St. Paul, USA; 4 Department of Forest Resources, University of Minnesota, St. Paul, USA; 5 Earth Sciences Division, Lawrence Berkeley National Laboratory, Berkeley, USA; 6 Department of Environmental Science and Engineering, Tsinghua University, Beijing, China

## Abstract

**Background:**

Elevated atmospheric CO_2_ (eCO_2_) has been shown to have significant effects on terrestrial ecosystems. However, little is known about its influence on the structure, composition, and functional potential of soil microbial communities, especially carbon (C) and nitrogen (N) cycling. A high-throughput functional gene array (GeoChip 3.0) was used to examine the composition, structure, and metabolic potential of soil microbial communities from a grassland field experiment after ten-year field exposure to ambient and elevated CO_2_ concentrations.

**Results:**

Distinct microbial communities were established under eCO_2_. The abundance of three key C fixation genes encoding ribulose-1,5-bisphosphate carboxylase/oxygenase (Rubisco), carbon monoxide dehydrogenase (CODH) and propionyl-CoA/acetyl-CoA carboxylase (PCC/ACC), significantly increased under eCO_2_, and so did some C degrading genes involved in starch, cellulose, and hemicellulose. Also, *nifH* and *nirS* involved in N cycling were significantly stimulated. In addition, based on variation partitioning analysis (VPA), the soil microbial community structure was largely shaped by direct and indirect eCO_2_-driven factors.

**Conclusions:**

These findings suggest that the soil microbial community structure and their ecosystem functioning for C and N cycling were altered dramatically at eCO_2_. This study provides new insights into our understanding of the feedback response of soil microbial communities to elevated CO_2_ and global change.

## Background

The concentrations of atmospheric CO_2_ have been increasing for the last 150 years and are predicted to increase to 550 ppm by the middle of this century [1]. This ongoing increase in atmospheric CO_2_ is due to the extensive use of fossil fuels and changes in land use patterns [2]. The rapid increase of CO_2_ in the atmosphere over the last century has led to an increase of global ecosystem carbon storage [3]. Terrestrial ecosystems are intimately connected to atmospheric CO_2_ levels and soil is the major organic C pool in all terrestrial biomes [4]. Studies of ecosystem responses to elevated CO_2_ have shown that eCO_2_ can have major effects on terrestrial ecosystems by enhancing plant photosynthetic CO_2_ fixation and primary productivity, and altered plant and soil characteristics [5-9]. However, the disparity between modeling and empirical studies suggests as yet incomplete understanding of the combined impacts of this global change factor on ecosystem functioning.

Since microorganisms mediate important biogeochemical processes such as soil C and N cycling, and are expected to influence future atmospheric CO_2_ concentrations, functional understanding of how eCO_2_ affects soil microbial community composition and structure will be necessary for robust prediction of atmospheric CO_2_ concentrations in the future. However, one of the major challenges for characterizing the functional diversity and their responses to the changes of atmospheric CO_2_ concentration is the extreme diversity and as-yet uncultivated status of many microorganisms. To date, most of the efforts to describe the effects of atmospheric CO_2_ concentration to soil microbial communities have been focused on phylogenetic composition [5,10,11]. Some studies [12,13] tried to examine the responses of soil microbial community to the changes of CO_2_ concentration. However, distinctly different results of the soil microbial diversity and activity under eCO_2_ have been obtained in different studies [11,14-17], and the possible relationships between the microbial community functional structure and the plant and soil parameters are still not clear.

Functional gene arrays (FGAs), such as GeoChip, which contain key genes encoding functional enzymes involved in biogeochemical cycling, have been successfully used for tracking and studying the biogeochemical processes in different ecosystems, including groundwater and aquatic ecosystems, soil, extreme environments, bioreactor systems, and oil-contaminated waters or soils [18,19]. Combined with multivariate statistical analyses [20], several systematic experimental evaluations have indicated that GeoChip can be used as a specific, sensitive tool for detecting the functional diversity, composition, structure, and metabolic potential of microbial communities, and correlating microbial communities to ecosystem processes and functioning [21-24].

We hypothesized that soil microbial community composition and structure would be altered directly or indirectly by eCO_2_, and that the functional gene groups involved in C and N cycling would be enhanced due to the increase of soil C input under eCO_2_[25]. To test those hypotheses, we conducted our experiments at the Cedar Creek Ecosystem Science Reserve in Minnesota (http://www.biocon.umn.edu/). A comprehensive functional gene array, GeoChip 3.0 [26], was used to analyze the function composition and structure of soil microbial communities under both ambient and elevated CO_2_ concentrations. Some key genes involved in C and N cycling were stimulated under CO_2_. This study provides new information for our understanding of the feedback response of soil microbial communities to eCO_2_.

## Results

### Overall responses of microbial C and N cycling genes under CO_2_

**Figure 1:**
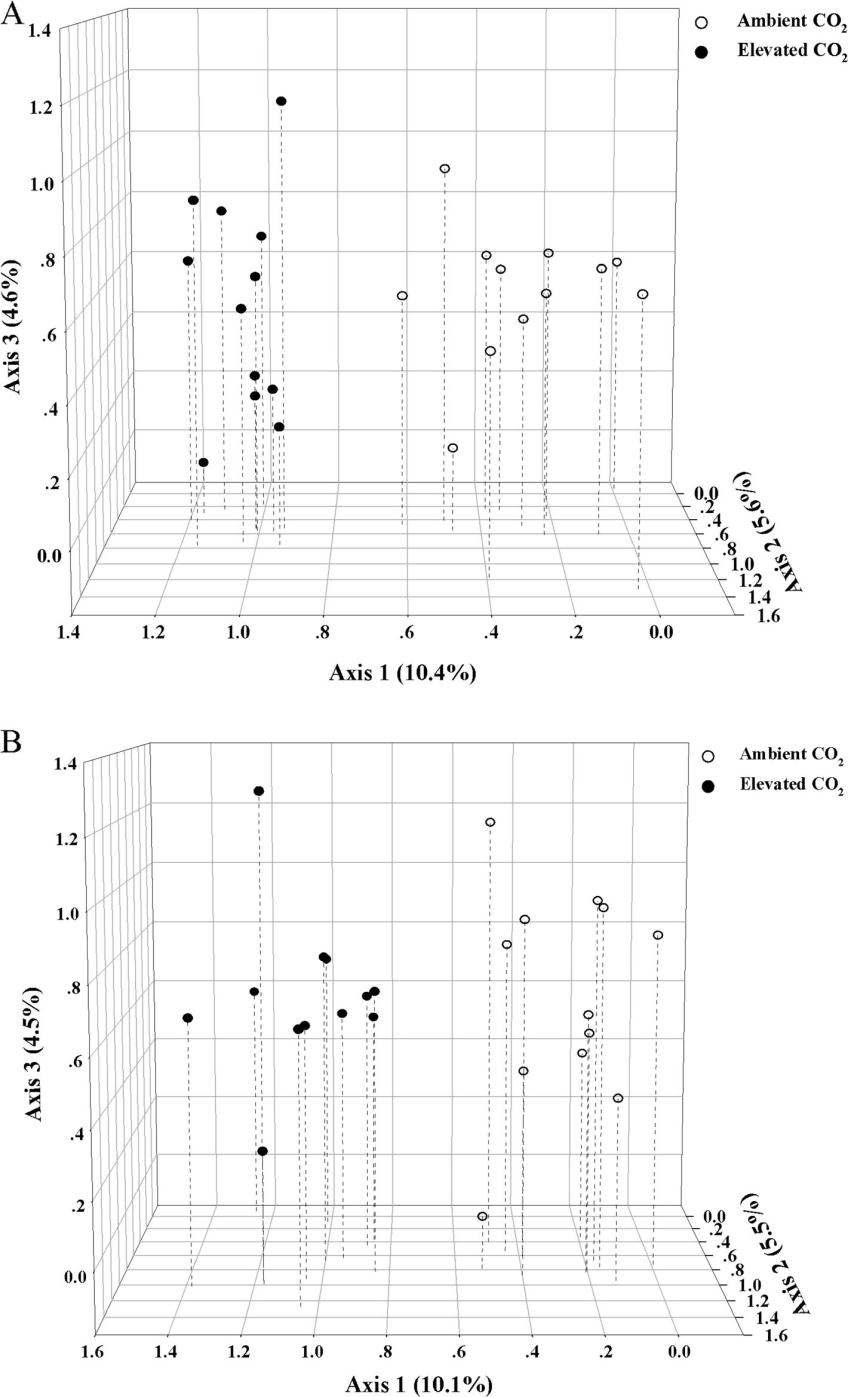
**Detrended correspondence analysis (DCA) of the samples under ambient and elevated CO**_**2 **_**bsed on GeoChip 3.0 data of the genes involved in carbon (A) and nitrogen (B) cycling.**

Based on the number of functional genes, Shannon diversity, evenness and dominance, no significant differences were detected in the overall microbial diversity (Additional file 1). Significant (*p* < 0.05) differences were observed in the abundance of C and N cycling genes between ambient CO_2_ (aCO_2_) and eCO_2_ microbial communities by detrended correspondence analysis (DCA) together with analysis of similarities (ANOSIM), non-parametric multivariate analysis of variance (Adonis) and Multi-Response Permutation Procedure (MRPP). The eCO_2_ samples were well separated from aCO_2_ ones by the first axis of DCA, which explained 10.4% and 10.1% for the genes involved in C cycling (Figure 1A) and N cycling (Figure 1B), respectively. These results suggest that most of the functional genes involved in C and N cycling were significantly stimulated, and that the functional composition and structure of soil microbial communities were also altered at eCO_2_. More details about individual key C and N cycling genes and their associated populations are described below.

### Responses of C cycling genes to eCO_2_

#### (i) Carbon fixation

Five pathways for autotrophic CO_2_ fixation have been identified [27]. Based on normalized signal intensities, 147 C fixation genes in four functional gene families were detected. Within this four functional gene families, two gene families encoding ribulose-1,5-bisphosphate carboxylase/oxygenase (Rubisco) and carbon monoxide dehydrogenase (CODH) significantly increased (*p* < 0.05), and another one encoding propionyl-CoA/acetyl-CoA carboxylase (PCC/ACC) showed increase trend at *p* < 0.1 level under eCO_2_. Individual gene variants and dominant populations about those three gene families were examined to understand the potential of microbial CO_2_ fixation in soil at eCO_2_.

**Figure 2:**
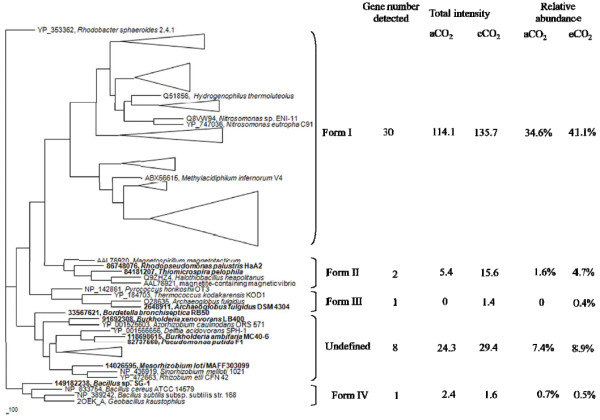
**Maximum-likelihood phylogenetic tree of the deduced amino acid sequences of Rubisco large subunit genes obtained from GeoChip 3.0, showing the phylogenetic relationship among the five Rubisco clusters.** The depth and width of each wedge is proportional to the branch lengths and number of Rubisco sequences, respectively. Some individual genes detected are shown in bold. The scale indicates the number of amino acid substitutions per site and the tree is outgroup rooted with YP_353362 (*Rhodobacter sphaeroides* 2.4.1).

So far, Rubisco has been classified into four forms [28]. A total of 46 *rbcL* probes encoding the large subunit of Rubisco had positive signals with 27 shared by both CO_2_ conditions, 8 and 11 unique at aCO_2_ and eCO_2_, respectively. All four forms of Rubisco were detected, but more than 70% of the gene variants belonged to Form I, especially for those significantly changed and dominant variants mentioned above. Only two genes belonged to Form II with one (84181207 from *Thiomicrospira pelophila*) unique to eCO_2_ and the other (86748076 from *Rhodopseudomonas palustris* HaA2) exhibiting increased signal intensity at eCO_2_. One eCO_2_ unique gene (2648911 from *Archaeoglobus fulgidus* DSM 4304) belonged to Form III and one unchanged gene (149182238 from *Bacillus* sp. SG-1) belonged to Form IV (Figure 2). In addition, eight variants detected were clustered as the undefined Form. No significant change was observed in these *rbcL* genes detected, except two showed increase trends and two showed decrease at *p* < 0.1 level under eCO_2_ (Additional file 2). For the other two gene families, two and six significant increase genes were detected in CODH (Additional file 3) and PCC (Additional file 4), respectively. Details for these gene variants and dominant populations are described in the Additional file 5.

#### (ii) Carbon degradation

GeoChip 3.0 targets many genes involved in labile C and recalcitrant C degradation. Overall, 429 C degradation genes in 24 functional gene families were detected and 26 genes showed significant (*p* < 0.05) changes with 15 increased and 11 decreased at eCO_2_ based on the signal intensity detected.

**Figure 3:**
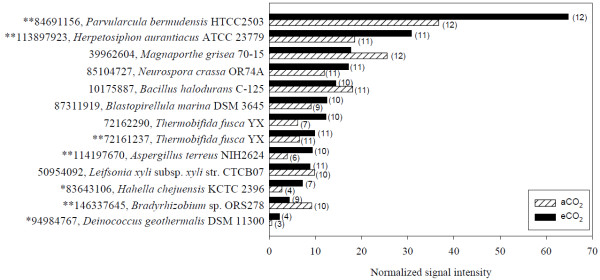
**The top ten abundant and other significantly changed *amyA *genes.** The number of the probes detected from eCO_2_ and aCO_2_ were presented following the bars in parentheses. The statistical significant results of response ratio were shown in front of the GenBank accession number of the probes (**p < 0.05, *p < 0.10).

Based on the normalized average signal intensity of key gene families detected among 12 soil samples under aCO_2_ or eCO_2_, the genes involved in hydrolysis of starch and other labile polysaccharides such as α-amylases (EC 3.2.1.1), glucoamylases (EC 3.2.1.3) and pullulanases (EC 3.2.1.41) significantly (*p* < 0.05) increased at eCO_2_. Among 68 detected *amyA* probes, 44 were shared by both CO_2_ conditions. For those shared genes, six gene variants showed strongly increasing trends with four genes (84691156 from *Parvularcula bermudensis* HTCC2503, 113897923 from *Herpetosiphon aurantiacus* ATCC 23779, 72161237 from *Thermobifida fusca* YX, and 114197670 from *Aspergillus terreus* NIH2624) at *p* < 0.05 level and two genes (83643106 from *Hahella chejuensis* KCTC 2396 and 94984767 from *Deinococcus geothermalis* DSM 11300) at *p* < 0.10 level, and one gene variant (146337645 from *Bradyrhizobium* sp. ORS278) showed significant decrease at *p* < 0.05 level at eCO_2_ (Figure 3). Within the 24 unique *amyA* genes, 11 were detected at aCO_2_ and 13 were detected at eCO_2_, and they contributed approximately 8.6% (3.4% for aCO_2_ and 5.2% for eCO_2_) of the total *amyA* signal intensity. The significant increase genes, 84691156 (from *Parvularcula bermudensis* HTCC2503) and 113897923 (from *Herpetosiphon aurantiacus* ATCC 23779), also ranked as the first and second abundant *amyA* genes with 13.2% and 7.7% of the total *amyA* gene signal, respectively (Figure 3). These results suggested that starch degradation by microorganisms in soil may increase at eCO_2_. Similar trends about the gene variants and dominant populations were observed in glucoamylase (Additional file 6) and pullulanase (Additional file 7). Details for these two gene families are described in Additional file 5.

Additionally, the abundance of key genes involved in the degradation of more complex C showed significantly increasing trends at eCO_2_, such as hemicellulose at *p* < 0.05 and cellulose at *p* < 0.1 level. For hemicellulose degradation, three gene families such as arabinofuranosidase (AFase, EC 3.2.1.55), cellobiase (EC 3.2.1.4) and xylanase (EC 3.2.1.8) were detected and the abundance of normalized signal intensity of AFase genes increased significantly (*p* < 0.05) in the normalized signal intensity under eCO_2_. The abundance of nine detected endoglucanase genes showed increases at *p* < 0.1 level under eCO_2_. Details regarding gene variants and dominant populations of endoglucanase (Additional file 8) and AFase (Additional file 9) genes are described in Additional file 5.

Finally, ten gene families encoding the enzymes for more complex or recalcitrant C degradation were detected with three for aromatic degradation (limonene-1,1-epoxide hydrolase, vanilate demethylase and vanillin dehydrogenase), three for chitin degradation (acetylglucosaminidase, endochitinase and exochitinase) and four for lignin degradation (glyoxal oxidase, lignin peroxidase, manganese peroxidase and phenol oxidase). However, based on the normalized signal intensity, only vanilate demethylase genes showed a significant increase (*p* < 0.05) under eCO_2_ (Additional file 10). The details about this gene are described in Additional file 5.

The above results clearly indicate that microbial CO_2_ fixation may increase, and that microbial degradation and utilization of labile C substrates (e.g., starch, cellulose) may also increase at eCO_2_, but the degradation of recalcitrant C (e.g., lignin) may not be stimulated by eCO_2_.

### Responses of N cycling genes to eCO_2_

Sixteen enzymes/genes involved in different N cycling processes were selected in GeoChip 3.0 to target important N cycling processes, such as N_2_ fixation, nitrification, and denitrification. Based on the total signal intensity detected, significant changes were observed in *nifH* and *nirS*, but not other N cycling genes.

N_2_ fixation is exclusively performed by prokaryotes, and *nifH* encoding the iron protein of N synthase complex, nitrogenase, is the most widely used functional gene marker for N_2_ fixation [29] and also a phylogenetic marker for *nifH*-containing organisms [30]. A total of 147 *nifH* gene variants were detected with 92 shared by both aCO_2_ and eCO_2_ samples, 41 unique to eCO_2_, and 15 unique to aCO_2_ samples. The total normalized signal intensity of these detected *nifH* genes was significantly (*p* < 0.05) higher at eCO_2_ than that at aCO_2_. Ten gene variants were significantly (*p* < 0.05) increased, and five were significantly decreased at eCO_2_. More than 69% of the *nifH* genes detected were affiliated with uncultured or unidentified microorganisms, and five (44829093, 12001884, 780709, 89512880, and 3157614) had >3.0% of the total *nifH* gene signal intensity.

**Figure 4:**
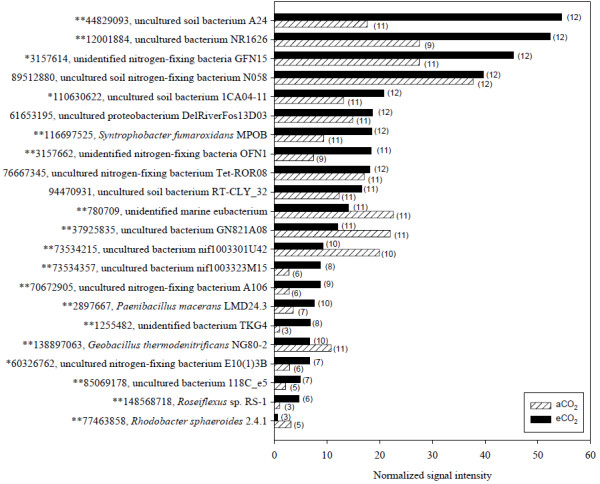
**The top ten abundant and other significantly changed *nifH *genes.** The number of the probes detected from eCO_2_ and aCO_2_ were presented following the bars in parentheses. The statistical significant results of response ratio were shown in front of the GenBank accession number of the probes (**p < 0.05, *p < 0.10).

For 13 significantly increased *nifH* gene variants, ten were from the uncultured or unidentified bacteria, and three (116697525, 2897667, and 148568718) were derived from *Syntrophobacter fumaroxidans* MPOB, *Paenibacillus macerans*, and *Roseiflexus* sp. RS-1, respectively. Similarly, for five significantly decreased genes detected, three were from unidentified marine eubacterium and unidentified bacteria, and two (77463858 and 138897063) were derived from *Rhodobacter sphaeroides* 2.4.1 and *Geobacillus thermodenitrificans* NG80-2, respectively (Figure 4). It is also noted that nine of the top ten abundant genes were from uncultured or unidentified bacteria (Figure 4).

**Figure 5:**
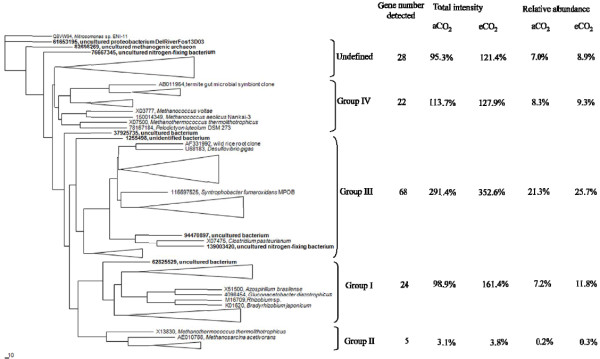
**Maximum-likelihood phylogenetic tree of the deduced amino acid sequences of nifH sequences obtained from GeoChip 3.0, showing the phylogenetic relationship among the five *nifH *clusters.** The depth and width of each wedge is proportional to the branch lengths and number of *nifH* sequences, respectively. Some individual genes detected are shown in bold. The scale indicates the number of amino acid substitutions per site and the tree is outgroup rooted with Q8VW94 (*Nitrosomonas* sp. ENI-11).

NifH has been classified into five distinct evolutionary groups [31]. Based on the sequences of *nifH* probes, the detected *nifH* genes were clustered into Group I - IV. Within these four groups, Group III had 68 *nifH* genes detected, and Groups I, IV, and II had 24, 22, and 5 genes detected, respectively. There were 28 *nifH* genes for the undefined group (Figure 5). In the major group (Group III), 21.3% and 25.7% relative abundances were detected from aCO_2_ and eCO_2_ samples, respectively. Similar signal intensity distributions were observed in Group I, Group IV and the undefined Group with 7.2%, 8.3% and 7.0% relative abundances from the aCO_2_ samples and 11.8%, 9.3% and 8.9% from the eCO_2_ samples, respectively. Within five genes in Group II, the relative abundances from the two aCO_2_ genes and the three eCO_2_ were 0.2% and 0.3%, respectively. Among these five groups, significant increase in the total signal intensity under eCO_2_ was only observed in Group I, although higher total signal intensities at eCO_2_ were detected in all five groups (Figure 5)_._

Among the 60 *nirS* genes detected, 31 were shared by both aCO_2_ and eCO_2_ samples (Additional file 11), whereas 23 and six were unique to eCO_2_ and aCO_2_, respectively (Additional file 12). Details for *nirS* gene are described in the Additional file 5. The above results indicate that N cycling may be significantly changed at eCO_2_, which was reflected in a significant increase in the abundance of detected *nifH* and *nirS* genes. Furthermore, the great *nirS* gene abundance would suggest the great N_2_O (a recognized greenhouse gas) emissions under eCO_2_ condition.

### Relationships between the microbial community structure and environmental factors

**Figure 6:**
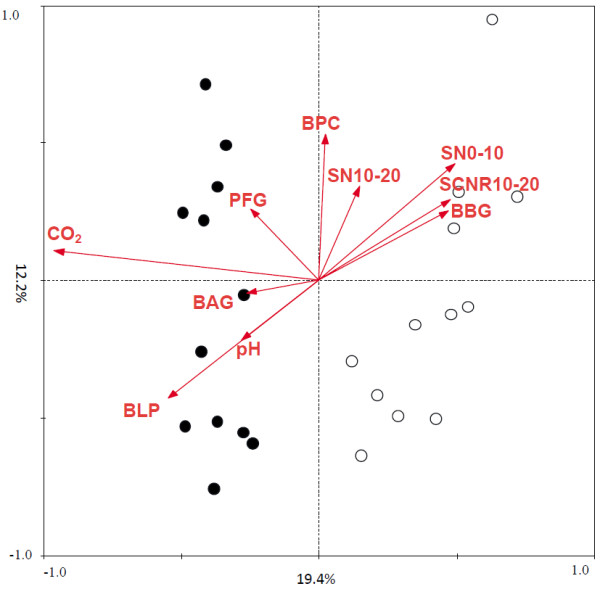
**Biplot of redundancy analysis (RDA) of entire functional gene communities of soil samples from aCO**_**2 **_**and eCO**_**2 **_**conditions.** Open circles represent samples collected from aCO_2_, whereas solid circles represent samples collected from eCO_2_. Four soil variables: soil N% at the depth of 0–10 ( SN0-10) and 10–20 cm (SN10-20), soil C and N ratio at the depth of 10–20 cm (SCNR10-20) and soil pH (pH), and five plant variables: biomass of C4 plant species *Andropogon gerardi* (BAG) and *Bouteloua gracilis* (BBG), biomass of legume plant species *Lupinus perennis* (BLP), below ground plant C percentage (BPC), and the number of plant functional groups (PFG), were selected by forward selection based variance inflation factor (VIF) with 999 Monte Carlo permutations.

The concentrations of atmospheric CO_2_ and nine environmental variables including four soil variables, soil N% at the depth of 0-10 cm (SN0-10) and 10–20 cm (SN10-20), soil C and N ratio at the depth of 10–20 cm (SCNR10-20), and soil pH (pH), and five plant variables, biomass of C4 plant species *Andropogon gerardi* (BAG) and *Bouteloua gracilis* (BBG), biomass of legume plant species *Lupinus perennis* (BLP), belowground plant C percentage (BPC), and the number of plant functional groups (PFG) were selected by forward selection based on variance inflation factor (VIF) with 999 Monte Carlo permutations. The VIF of these ten parameters were all less than 6.5. Although the rates of biogeochemical processes about nitrification, ammonification and net N mineralization were also detected, these three parameters were rejected by forward selection since their VIF were all higher than 100. The relationships between the functional structure of soil microbial communities and the ten parameters selected were analyzed by redundancy analysis (RDA) and the ordination plot (Figure 6) was very consistent with the DCA ordination patterns. The samples from aCO_2_ and eCO_2_ were well separated by the first axis of RDA with 19.4% explained by the first axis and a total of 47.6% explained with microbial communities (*p* = 0.047). Similar RDA results were obtained for subsets of functional genes, with 48.1% of the total variance explained for the C cycling genes (*p* = 0.037) and 48.2% of the total variance explained for the N cycling genes (*p* = 0.044). Within these variables, all detected functional genes and subsets of those genes were significantly different between CO_2_ treatments (*p* = 0.001).

To better understand the relationships between the functional structure of soil microbial communities and the plant and soil variables, variation partitioning analysis (VPA) was performed. After accounting for the effects of the CO_2_ treatment, the nine environmental variables could explain 42.2%, 42.8% and 42.8% of the total variation for all detected genes (*p* = 0.098), C cycling genes (*p* = 0.072), and N cycling genes (*p* = 0.087), respectively (Table 1). These five selected plant variables could significantly explain 24.7% (*p* = 0.010) of the variance for all detected genes, 24.6% (*p* = 0.022) for detected C cycling genes, and 25.1% (*p* = 0.014) for detected N cycling genes (Table 1). For the soil variables, these four selected variables also could explain 19.4% (*p* = 0.053) of the variance for all detected genes, 19.0% (*p* = 0.146) for detected C cycling genes, and 19.7% (*p* = 0.067) for detected N cycling genes (Table 1). Within these nine selected parameters, distinct differences were observed between the samples from aCO_2_ and eCO_2_ (*p* values ranged from 0.023 to 0.092), and the variance explained by four of the important variables, including pH (*r* = 0.411, *p* = 0.046), BLP (*r* = 0.378, *p* = 0.069), BPC (*r* = −0.345, *p* = 0.098), and PFG (*r* = 0.385, *p* = 0.063).

**Table 1:** **The relationships of microbial community functional structure to plant and soil characteristics by RDA and VPA**^***a***^

		**All genes detected**	**C cycling genes**	**N cycling genes**
With nine selected variables	First axis explanation (%)	19.1	20.3	19.6
	Total explanation (%)	42.2	42.8	42.8
	*F*	1.138	1.167	1.163
	*p*	0.098	0.072	0.087
Explanations of the selected plant variables (%)	Total	24.7	24.6	25.1
	The number of plant functional groups (PFG)	5.9	4.5	5.1
	Belowground plant C percentage (BPC)	4.4	4.5	4.5
	Biomass of C4 plant species *Andropogon gerardi* (BAG)	4.4	3.7	4.5
	Biomass of C4 plant species *Bouteloua gracilis* (BBG)	3.7	4.5	3.8
	Biomass of legume plant species *Lupinus perennis* (BLP)	6.0	6.0	6.4
Explanations of the selected soil variables (%)	Total	19.4	19.0	19.7
	Soil N% at the depth of 0-10 cm (SN0-10)	5.7	5.2	4.5
	Soil N% at the depth of 10-20 cm (SN10-20)	4.4	4.5	5.1
	Soil C and N ratio at the depth of 10–20 cm (SCNR10-20)	4.4	4.5	3.8
	pH	4.4	5.2	5.1

^*a*^ The covariables for plant and soil variables were close zero.

## Discussion

It is hypothesized that eCO_2_ may affect soil microbial C and N cycling due to the stimulation of plant photosynthesis, growth, and C allocation belowground [25,32,33] . Previous studies from the BioCON experiment showed that eCO_2_ led to changes in soil microbial biomass, community structure, functional activities [13,34,35], soil properties, such as pH and moisture [36], and microbial interactions [37]. Also, another study with Mojave Desert soils indicated that eCO_2_ increased microbial use of C substrates [17]. Consistently, our GeoChip data showed that the composition and structure of functional genes involved in C cycling dramatically shifted with a general increase in abundance at eCO_2_. First, this is reflected in an increase of abundances of microbial C fixation genes. Three key C fixation genes increased significantly at eCO_2_, including Rubisco for the Calvin–Benson–Bassham (CBB) cycle [38], CODH for the reductive acetyl-CoA pathway [39], and PCC/ACC for the 3-hydroxypropionate/malyl-CoA cycle [40]. It is expected that Form II Rubiscos would be favored at high CO_2_ and low O_2_ based on the kinetic properties [28]. Indeed, two Form II Rubiscos genes from *Thiomicrospira pelophila* (γ-Proteobacteria) and *Rhodopseudomonas palustris* HaA2 (α-Proteobacteria) were unique or increased at eCO_2_, respectively. For *Thiomicrospira*, the Form II Rubiscos are presumably expressed in the more anaerobic environments at high CO_2_[28], while *R*. *palustris* has extremely flexible metabolic characteristics including CO_2_ and N_2_ fixation under anaerobic and phototrophic conditions [41]. The second most abundant CODH gene was also detected from *R*. *palustris* and increased significantly at eCO_2_, and its dominant populations were found to be acetogenic bacteria, which may function for converting CO_2_ to biomass under anaerobic conditions. Since the knowledge of microbial C fixation processes in soil is still limited, mechanisms of the response of microbial C fixation genes to eCO_2_ need further study. Second, significant increases were observed in the abundance of genes involved in degradation of labile C substrates, such as the genes encoding α-amylase, glucoamylase and pullulanase for starch degradation, arabinofuranosidase for hemicellulose degradation, and endoglucanase for cellulose degradation. However, no significant change was shown in the abundance of genes involved in recalcitrant C (e.g., lignin) degradation. Therefore, our results indicate that eCO_2_ significantly affected metabolic potentials for C fixation and degradation. However, it appears that such changes have little effect on soil C storage [25], probably due to accelerated degradation of increased C inputs, which is consistent with increased soil CO_2_ flux over the course of the experiment.

Another important question is whether eCO_2_ affects soil N cycling processes and/or soil N dynamics. Our previous study has showed that soil N supply is probably an important constraint on global terrestrial productivity in response to eCO_2_[32]. When N is limiting, decomposers may respond to increased C inputs by decomposing soil organic matter to gain access to N and constrain the plant biomass accumulation at eCO_2_[42,43]. In this study, our GeoChip analysis showed that the abundance of *nifH* genes significantly increased at eCO_2_. Presumably, an increase in N_2_ fixation under eCO_2_ may lead to enhanced CO_2_ fertilization of plant biomass production by alleviating some of the N constraints on plant response to eCO_2_. In the plots examined in the present study, no N fertilizer was supplemented, but significant increases were observed in the total plant biomass and aboveground plant biomass, especially the biomass of legume plant species *Lupinus perennis*, which may be associated with significant increases of N_2_ fixers in soil under eCO_2_ measured by the abundance of *nifH* genes in this study. At eCO_2_, if the increased *nifH* abundance is interpreted as potential increase of soil microbial N_2_ fixation, such increase could supplement N nutrients for the plant growth to eliminate the N limitation constraint. In addition, the abundance of *nirS* genes significantly increased at eCO_2_ while all others genes involved in denitrification remained unaffected. The results suggest that eCO_2_ could significantly impact microbial N_2_ fixation and denitrification, and probably enhance the production of the greenhouse gas N_2_O. However, it appears that no significant changes were observed in soil N dynamics under eCO_2_, which may be largely due to the large N pool size in soil.

It is largely unknown whether or how eCO_2_ and eCO_2_-induced effects, such as increased C inputs into soil and changes in soil properties, shape soil microbial community structure. The direct effects of elevated atmospheric CO_2_ concentration on soil microbial communities were expected to be negligible compared to potential indirect effects such as increased plant C inputs to soil, since the CO_2_ concentrations in the pore space of soil generally is much higher than those in the atmosphere even under ambient CO_2_ concentrations [5]. However, this has not been well studied. Based on our GeoChip data, VPA showed the CO_2_ treatment could significantly explain 8.9% of the total variation of microbial community structure, 9.6% of detected functional genes involved in C cycling, and 9.4% of detected functional genes in N cycling in this study. After accounting for the effects of the CO_2_ treatment, the selected variables from plant and soil could significantly explain more than 42% of the total variances of microbial community structure. Our previous studies have demonstrated that increased C inputs at eCO_2_ stimulate microbial activity and regulate their composition [13,25]. Consistently, our statistical analysis suggests that the biomass of N_2_-fixing legume species (BLP) and the number of plant functional groups (PFG) have significantly positive correlations with the atmospheric CO_2_ level. These strong correlations could arise because increased plant-derived substrates at eCO_2_ could fuel heterotrophic metabolism in soil [44]. Such a strong correlation with the biomass of N_2_-fixing legume species (BLP) may result in an increased amount of N derived from the atmosphere. Therefore, significant increases in plant biomass were associated with the significant increase in the abundance of *nifH* genes, but little effect was seen in soil N dynamics.

Soil microbial community structure may be shaped by soil properties, such as pH and moisture [45]. For example, soil pH and moisture changed at eCO_2_ in the BioCON study [6,46], and a significant correlation between the soil microbial community compositions and soil pH was observed with a survey of 88 soils across North and South America [47]. In this study, soil N% at the depth of 0-10 cm (SN0-10) and 10–20 cm (SN10-20), soil C and N ratio at the depth of 10–20 cm (SCNR10-20), and soil pH (pH) were identified as the most important soil factors shaping microbial community structures. In addition, significant correlations were also observed between the plant and soil factors, such as positive correlations between pH and BBG, pH and PFG, SCNR10-20 and BBG, and negative correlations between SCNR10-20 and BLP. These results suggested that, in addition to direct effects of atmospheric CO_2_ on soil microbial C and N cycling, such as CO_2_ fixation, eCO_2_-induced indirect effects on plant and soil properties significantly impact the soil microbial community structure and modify their ecosystem functioning. The simultaneous enhances in the processes involved in CO_2_ fixation, C degradation, N fixations and partial denitrification could be the reason that no significant difference was detected in total soil C and N.

## Conclusions

GeoChip was successfully used to illuminate the response of soil microbial communities to eCO_2_. The results showed that microbial C and N cycling were altered dramatically at eCO_2_, and the eCO_2_-induced effects, such as increased plant biomass and altered soil pH, may largely shape the soil microbial community structure and regulate their ecosystem functioning. However, the impact of these changes on soil C and N dynamics need to be further investigated. This study provides important insights into our understanding of the feedback response of soil microbial communities to elevated CO_2_ and global change.

## Methods

### Site, sampling and environmental variable analysis

This study was conducted within the BioCON experiment site [6] located at the Cedar Creek Ecosystem Science Reserve, MN, USA. The main BioCON field experiment has 296 plots (2 by 2 m) in six 20-meter-diameter rings, three for an aCO_2_ concentration of 368 μmol/mol and three for an elevated CO_2_ concentration of 560 μmol/mol using a FACE system as described by Reich et al. [6]. In this study, soil samples without plant root from 24 plots (12 biological replicates from ambient CO_2_ and 12 biological replicates from elevated CO_2_. All with 16 native plant species including four C4 grasses, four C3 grasses, four N-fixing legumes and four non-N-fixing herbaceous species, and no additional N supply) were collected in July 2007. The aboveground and belowground biomass, plant C and N concentrations, soil parameters, and *in situ* net N mineralization and net nitrification were measured as previously described [6,32]. More detailed information about sampling is provided in Additional file 13.

### GeoChip analysis

DNA extraction, amplification and labeling, as well as the purification of labeled DNA, were carried out according the methods described by Xu *et al*. [23]. GeoChip 3.0 [26] was used to analyze the functional structure of the soil microbial communities. Details for GeoChip hybridization, image processing and data pre-processing are described in Additional file 13.

### Statistical analysis

Pre-processed GeoChip data were further analyzed with different statistical methods: (i) detrended correspondence analysis (DCA) [48], combined with analysis of similarities (ANOSIM), non-parametric multivariate analysis of variance (Adonis) and Multi-Response Permutation Procedure (MRPP), for determining the overall functional changes in the microbial communities; (ii) microbial diversity index, Significant Pearson’s linear correlation (*r*) analysis, analyses of variance (ANOVA) and response ratio (RR) [3]; (iii) redundancy analysis (RDA) for revealing the individual or set of environmental variables that significantly explained the variation in functional microbial communities; (iv) variation partitioning for RDA were used to select the minimum number of environmental variables explaining the largest amount of variation in the model [20,49]. More details about the data analysis are described in Additional file 13.

## Competing interests

The authors have declared that no competing interests exist.

## Authors’ contributions

Conceived and designed the experiments: MX, ZH, SEH, PBR and JZ. MX, LW, JDN performed the experiments. MX, ZH and DY analyzed the data. MX, ZH and JZ interpreted the data. MX and ZH drafted the manuscript. SEH, PBR and JZ were involved in editing and revising the manuscript critically in preparation for submission. All authors read and approved the final manuscript.

## Supplementary Material

Additional file 1 **A table listing the overall microbial community diversity detected by GeoChip under ambient CO**_**2 **_**(aCO**_**2**_) **and elevated CO**_**2 **_**(eCO**_**2**_**).** Click here for file

Additional file 2 **A figure about the normalized signal intensities of *rbcL *gene detected.** Click here for file

Additional file 3 A figure about the normalized signal intensities of CODH gene detected. Click here for file

Additional file 4 **A figure about the significantly changed and other top ten abundant *pcc *genes.** Click here for file

Additional file 5 **The supplemental results about the responses of carbon and nitrogen cycling genes to eCO**_**2**_**.** Click here for file

Additional file 6 A figure about the normalized signal intensities of glucoamylase encoding gene detected. Click here for file

Additional file 7 **A figure about the normalized signal intensities of *pulA *gene detected.** Click here for file

Additional file 8 A figure about the normalized signal intensities of endoglucanase gene detected. Click here for file

Additional file 9 **A figure about the normalized signal intensities of *ara *gene detected.** Click here for file

Additional file 10 **A figure about the normalized signal intensities of *vanA *gene detected.** Click here for file

Additional file 11 **A figure about the normalized signal intensities of shared *nirS *gene detected.** Click here for file

Additional file 12 **A table listing the *nirS *genes only detected at aCO**_**2 **_**or eCO**_**2**_**.** Click here for file

Additional file 13 The supplemental descriptions for materials and methods. Click here for file
