# Supplementary material for: Elevated CO2 influences microbial carbon and nitrogen cycling
Source: BMC Microbiol. 2013 May 29;13:124. doi: 10.1186/1471-2180-13-124 (PMC3679978; doi:10.1186/1471-2180-13-124)
Supplement: Additional file 1 — A table listing the overall microbial community diversity detected by GeoChip under ambient CO2 (aCO2) and elevated CO2 (eCO2). [file 1471-2180-13-124-S1.docx]

**Additional file 1.** Overall microbial community diversity detected by GeoChip under ambient CO_2_ (aCO_2_) and elevated CO_2_ (eCO_2_).

|  | *1/D* | *H*' | *Evenness* |
| --- | --- | --- | --- |
| aCO2 | 1811.70±271.64 | 11.66±0.14 | 0.29±0.03 |
| eCO2 | 1778.23±179.17 | 11.79±0.08 | 0.31±0.02 |

The microbial diversity indices, including *1/D*, *H’*, and *Evenness* were calculated using Krebs/win version 0.94 (<http://www.biology.ualberta.ca/jbrzusto/krebswin.html>).
