# Supplementary material for: Elevated CO2 influences microbial carbon and nitrogen cycling
Source: BMC Microbiol. 2013 May 29;13:124. doi: 10.1186/1471-2180-13-124 (PMC3679978; doi:10.1186/1471-2180-13-124)
Supplement: Additional file 2 — A figure about the normalized signal intensities of rbcL gene detected. [file 1471-2180-13-124-S2.doc]

(8)

(3)

(7)

(8)

(4)

(7)

(8)

(8)

(3)

(3)

(3)

(7)

(8)

(5)

(3)

(3)

(3)

(3)

(11)

(11)

(12)

(10)

(6)

(8)

(6)

(9)

(7)

(10)

(10)

(7)

(3)

(7)

(6)

(7)

(5)

(3)

(3)

(5)

(3)

(3)

(4)

(3)

(3)

(3)

(3)

(3)

(5)

(3)

(3)

(3)

(4)

(4)

(3)

(3)

(3)

(3)

(3)

(3)

(3)

(4)

(3)

(3)

(4)

(5)

(5)

(4)

(3)

(3)

(6)

(5)

(4)

**Additional file 2.** The normalized signal intensities of *rbcL* gene detected. The sample numbers detected from eCO_2_ and aCO_2_ for the probes were presented following the bars in parentheses. The statistical significant results of response ratio were shown in front of the GenBank accession number of the probes (**P* < 0.10).

| 91690340, *Burkholderia xenovorans* LB400 |
| --- |
| 89241998, *Symploca atlantica* PCC 8002 |
| *157064951, *Phormidium laminosum* 'OH-1-p Cl 1' |
| 86748076, *Rhodopseudomonas palustris* HaA2 |
| 157679103, *Acidithiobacillus thiooxidans* |
| 89241914, uncultured bacterium OT-cbbL1.31 |
| 118435893, *Stappia aggregata* IAM 12614 |
| *14026595, *Mesorhizobium loti* MAFF303099 |
| *91692308, *Burkholderia xenovorans* LB400 |
| *150014764, uncultured alpha proteobacterium Z843 |
| 88919941, *Acidiphilium cryptum* JF-5 |
| 157679123, uncultured bacterium YL5 |
| 22415761, *Synechocystis trididemni* |
| 91802339, *Nitrobacter hamburgensis* X14 |
| 118698615, *Burkholderia ambifaria* MC40-6 |
| 114320324, *Alkalilimnicola ehrlichei* MLHE-1 |
| 60475992, uncultured bacterium fg8L134 |
| 119964666, *Sulfobacillus acidophilus* |
| 60475948, uncultured bacterium fg1L583 |
| 157741864, *Mycobacterium* sp. DSM 3803 |
| 89362129, *Xanthobacter autotrophicus* Py2 |
| 154160944, *Xanthobacter autotrophicus* Py2 |
| 124485383, *Methanocorpusculum labreanum* Z |
| 60476006, uncultured bacterium fg10L476 |
| 149182238, *Bacillus* sp. SG-1 |
| 50542690, uncultured bacterium HSMR12 |
| 4160592, *Bradyrhizobium japonicum* |
| 84181207, *Thiomicrospira pelophila* |
| 33567621, *Bordetella bronchiseptica* RB50 |
| 114705666, *Fulvimarina pelagi* HTCC2506 |
| 50542742, uncultured bacterium HSMR139 |
| 89275899, uncultured gamma proteobacterium 47 |
| 90104852, *Rhodopseudomonas palustris* BisB18 |
| 124389563, *Nitrococcus mobilis* |
| 4836660, *Acidithiobacillus ferrooxidans* |
| 82737569, *Pseudomonas putida* F1 |
| 148254105, *Bradyrhizobium* sp. BTAi1 |
| 2648911, *Archaeoglobus fulgidus* DSM 4304 |
| 89241912, uncultured bacterium OT-cbbL1.26 |
| 21217709, uncultured bacterium ZZ17C12 |
| 47115400, *Oligotropha carboxidovorans* |
| 1101742, *Prochloron* sp. |
| 60476050, uncultured bacterium ng8L742 |
| 60475994, uncultured bacterium fg8L136 |
| 47680331, uncultured bacterium R45c |
| 32307669, uncultured bacterium ML2J-19R |

(6)

(8)
