# Supplementary material for: Elevated CO2 influences microbial carbon and nitrogen cycling
Source: BMC Microbiol. 2013 May 29;13:124. doi: 10.1186/1471-2180-13-124 (PMC3679978; doi:10.1186/1471-2180-13-124)
Supplement: Additional file 3 — A figure about the normalized signal intensities of CODH gene detected. [file 1471-2180-13-124-S3.doc]

**

**

67933455, Solibacter usitatus Ellin607677700893, Rhodopseudomonas palustris BisA5373748499, Dehalococcoides sp. CBDB177690377, Rhodopseudomonas palustris BisB586573243, Rhodopseudomonas palustris HaA268182286, Jannaschia sp. CCS186750309, Rhodopseudomonas palustris HaA271368294, Nocardioides sp. JS61467910920, Polaromonas sp. JS66667850650, Clostridium thermocellum ATCC 2740578517773, Bradyrhizobium sp. BTAi168190972, Mesorhizobium sp. BNC178518845, Bradyrhizobium sp. BTAi183575217, Rhodospirillum rubrum ATCC 1117086751065, Rhodopseudomonas palustris HaA286571765, Rhodopseudomonas palustris HaA284392538, Oceanicola batsensis HTCC2597

**Additional file 3.** The normalized signal intensities of CODH gene detected. ***P* < 0.05.
