# Supplementary material for: Elevated CO2 influences microbial carbon and nitrogen cycling
Source: BMC Microbiol. 2013 May 29;13:124. doi: 10.1186/1471-2180-13-124 (PMC3679978; doi:10.1186/1471-2180-13-124)
Supplement: Additional file 4 — A figure about the significantly changed and other top ten abundant pcc genes. [file 1471-2180-13-124-S4.doc]

**

**

**

**

*

*

**

**

*

121607822, *Verminephrobacter eiseniae* EF01-292117653, *Nitrobacter hamburgensis* X14148657926, *Roseiflexus* sp. RS-1106771910, *Roseiflexus* sp. RS-186569029, *Frankia* sp. CcI386357847, *Rhizobium* etli CFN 42149819232, *Plesiocystis pacifica* SIR-198977415, *Sphingopyxis alaskensis* RB2256158330288, *Azorhizobium caulinodans* ORS 57117937293, *Agrobacterium tumefaciens* str. C58117927612, *Acidothermus cellulolyticus* 11B108798265, *Mycobacterium* sp. MCS92443198, *Mycobacterium* sp. KMS90198261, *Mycobacterium vanbaalenii* PYR-183816321, *Salinibacter ruber* DSM 13855113731760, *Caulobacter* sp. K312634826, *Bacillus subtilis* subsp. *subtilis* str. 168

**Additional file 4** Significantly changed and other top ten abundant *pcc* genes. ***P* < 0.05, **P* < 0.10.
