# Supplementary material for: Elevated CO2 influences microbial carbon and nitrogen cycling
Source: BMC Microbiol. 2013 May 29;13:124. doi: 10.1186/1471-2180-13-124 (PMC3679978; doi:10.1186/1471-2180-13-124)
Supplement: Additional file 5 — The supplemental results about the responses of carbon and nitrogen cycling genes to eCO2. [file 1471-2180-13-124-S5.docx]

**Additional file 5. Supplemental Results**

**1. Responses of carbon cycling genes to eCO_2_**

*(i) Carbon fixation*

A total of 46 *rbcL* probes encoding the large subunit of Rubisco had positive signals with 27 shared by both CO_2_ conditions, 8 and 11 unique at aCO_2_ and eCO_2_, respectively. Among these shared probes, two (150014764 from an uncultured *α-Proteobacterium* and 157064951 from *Phormidium laminosum* OH-1-p Cl 1) showed significant increases (Additional file 2). These unique variants detected at aCO_2_ or eCO_2_ contributed approximately 17.4% (6.9% for aCO_2_ and 10.4% for eCO_2_) of the total *rbcL* signal intensity detected, and they were generally detected in 3-5 samples among the 12 aCO_2_ or 12 eCO_2_ samples. Based on signal intensity, the most abundant *rbcL* variant was 91690340 from *Burkholderia xenovorans* LB400, and together with other two variants, 91692308 and 118698615 from the same species and genus, respectively, *Burkholderia* species appeared to be the dominant populations with approximately 20% of the total *rbcL* signal intensity detected, although only 91692308 showed change at *p*<0.1 at eCO_2_ (Additional file 2). The second abundant variant was 89241998 from *Symploca atlantica* PCC 8002 with about 5.3% of the total *rbcL* signal intensity, but it did not significantly change at eCO_2_ (Additional file 2). The third abundant variant with 5.0% of the total *rbcL* signal intensity was 157064951 from *Phormidium laminosum* OH-1-p Cl 1 that showed a increase trend (*p*<0.1) at eCO_2_ (Additional file 2).

Among 17 CODH genes detected, nine of them were shared by both aCO_2_ and eCO_2_ samples with two genes (77700893 and 67933455) significantly (*p*<0.05) increased at eCO_2_, but no genes showed a significant decrease at eCO_2_ based on their signal intensities (Additional file 3). Four and four CODH genes were only detected at aCO_2_ or eCO_2_, respectively, and those unique variants contributed approximately 9.9% (4.1% for aCO_2_ and 5.8% for eCO_2_) of the total CODH signal intensity detected, which were detected in 3-6 samples among 12 aCO_2_ or eCO_2_ samples. Three most abundant CODH genes were detected to be 67933455 derived from *Solibacter usitatus* Ellin6076, 77700893 from *Rhodopseudomonas palustris* BisA53, and 73748499 from *Dehalococcoides* sp. CBDB1 with about 30%, 19.6%, and 13.8% of the total CODH gene signal intensity detected, respectively. The signal intensity of the first two genes significantly (*p*<0.05) increased at eCO_2_ although the third did not show a significantly change (Additional file 3).

For Pcc genes, 79 probes showed positive signals, and 45 were shared by both aCO_2_ and eCO_2_ samples, while 10 and 24 were only detected at aCO_2_ and eCO_2_, respectively. Among the shared 45 genes, six gene variants showed significant (*p* < 0.05) changes with five genes (117927612, 17937293, 118173204, 149819232, and 121607822) increased and one gene (120589257) decreased at eCO_2_ based on the signal intensity. At the *p* < 0.1 level, additional three genes showed significant changes with an increase for one (106771910), and a decrease for two genes (118045694, and 111611644) at eCO_2_ (Additional file 4). Those 34 unique variants contributed approximately 12.0% of the total Pcc gene signal intensity detected with 3.8% for aCO_2_ and 8.2% for eCO_2_. The most abundant Pcc gene was 121607822 derived from *Verminephrobacter eiseniae* EF01-2, a β-Proteobacterium with 13.7% of the total Pcc gene signal intensity and significantly (*p*<0.05) increased at eCO_2_ (Additional file 4). Another three Pcc genes (111611107, 121553501, and 111611644) were also detected for this strain with 5.9% of the total Pcc gene signal intensity. *V. eiseniae* is associated with earthworm nephridia in a poorly understood animal-bacterium symbiotic system (<http://genome.jgi-psf.org/mic_home.html>). The second abundant Pcc gene was 92117653 from an α-Proteobacterium, *Nitrobacter hamburgensis* X14 with 7.5% of the total Pcc gene signal intensity but without a significant change at eCO_2_ (Additional file 4). *Nitrobacter hamburgensis* is a common soil bacterium, which is not only able to fix CO_2_, but also to oxidize nitrite [1]. The third and forth abundant Pcc genes (148657926 and 106771910) were all from *Roseiflexus* sp. RS-1 with 6.6% and 4.5% of the total Pcc gene signal intensity respectively. *Roseiflexus* sp. RS-1 belongs to the Chloroflexi phylum, and *Roseiflexus* species appear to have the capacity for autotrophic CO_2_ fixation [2]. The last three most abundant Pcc genes did not show significant changes at eCO_2_ (Additional file 4).

*(ii) Carbon degradation*

Glucoamylases hydrolyze starch and other polysaccharides. Overall, glucoamylase genes showed a significant increase (*p*<0.05) in signal intensity under eCO_2_. Ten Glucoamylase probes showed positive signals with seven shared by both CO_2_ conditions, one and two appeared to be unique to aCO_2_ and eCO_2_, respectively. For those shared gene variants, two (150377998 from *Sinorhizobium medicae* WSM419 and 67157834 from *Azotobacter vinelandii* AvOP) significantly (*p*<0.10) increased at eCO_2_ based on signal intensity (Additional file 6). The shared genes had 97.1% of the total Glucoamylase gene signal intensity, and the unique genes had 2.9% (1.1% for aCO_2_, and 1.8% for eCO_2_) (Additional file 6), further suggesting the shared genes/populations may play more important roles than unique genes/populations in soil microbial communities in response to eCO_2_. The first and second most abundant probes target 150377998 from *Sinorhizobium medicae* WSM419 and 67157834 from *Azotobacter vinelandii* AvOP with 23.7% and 20.6% of the total Glucoamylase gene signal, respectively, which also showed significant (*p*<0.10) increase at eCO_2_ (Additional file 6). *S. medicae* WSM419 belongs to α-Proteobacteria isolated from *Medicago truncatula*, where it forms nitrogen-fixing root nodules [3]. Similarly, pullulanases hydrolyze starch and other polysaccharides with starch-debranching activity [4]. *pulA* probes showed a significant increase (*p*<0.05) in signal intensity under eCO_2_. The shared genes had 91.3% of the total *pulA* signal intensity, and the unique genes had 8.7% (1.9% for aCO_2_ and 6.8% for eCO_2_. Unique genes were detected in 3 of 12 samples at aCO_2_, and 3-6 of 12 samples at eCO_2_ (Additional file 7).

Within the nine genes encoding endoglucanase for cellulose degradation, five shared by both CO_2_ conditions, four unique to eCO_2_, and no genes detected at aCO_2_ (Additional file 8). The shared genes had 80.6% of the total endoglucanase gene signal intensity with one individual shared genes showed a significant (*p*<0.10) change at eCO_2_. The unique genes detected in 3-6 samples of 12 at eCO_2_ had 19.4% of the total signal (Additional file 8). The most abundant probe for endoglucanase genes was 55295400 from *Irpex lacteus* with 30.5% of the total signal of detected endoglucanase genes. *I. lacteus* is a basidiomycete fungus and produces cellulases, such as exoglucanases (or cellobiohydrolase) and endoglucanases for cellulose degradation (Toda et al., 2005 BBB). Two probes for endoglucanase genes (31747164 and 77176916) were detected in *Hypocrea jecorina* with 17.2% and 16.5% of the total signal of detected endo-glucanase genes, respectively and the gene 31747164 showed significant increase at *p* < 0.10 under eCO_2_. *H. jecorina* is now known as *Trichoderma reesei*, a filamentous fungus widely used in industry for cellulase and hemicellulase production, and recent genome sequence analyses show that numerous genes encoding biosynthetic pathways for secondary metabolites may promote survival of *T. reesei* in its competitive soil habitat [5]. The third most abundant probe targets 66845524 (*Aspergillus fumigatus* Af293) with 8.3% of the total endoglucanase gene signal. The genus *Aspergillus* is found worldwide and consists of more than 180 officially recognized species, and comprises a particularly important group of filamentous ascomycete species. Most of the *Aspergillus* members are useful microorganisms in nature for degradation of plant polysaccharides, and they are important industrial microorganisms for the large-scale production of both homologous and heterologous enzymes [6].

AFases degrade hemicellulose with L-arabinofuranosidic linkages and releases arabinose in an exo-manner from substrates such as arabinoxylan and arabinan [7]. The abundance of normalized signal intensity of AFase genes showed a significant increase (*p*<0.05) in relative signal intensity under eCO_2_. Thirty-six individual AFase genes showed positive signals with 24 shared by both CO_2_ conditions, five and seven appeared to be unique to aCO_2_ and eCO_2_, respectively (Additional file 9). Among the shared genes, four genes showed significant increases with two genes (113733431 from *Caulobacter* sp. K31 and 21220080 from *Streptomyces coelicolor* A3) at *p*<0.05 level, and two genes (106889690 from *Roseiflexus* sp. RS-1 and 156934116 from *Enterobacter sakazakii* ATCC BAA-894) showed a significant increase under eCO_2_ at *p* < 0.1. The shared genes and the unique genes had 89.8% and 10.2% (3.8% for aCO_2_, and 6.4% for eCO_2_) of the total AFase gene signal intensity. The first and second most abundant probes target 113733431 from *Caulobacter* sp. K31 and 106889690 from *Roseiflexus* sp. RS-1 with 12.6% and 8.5% of the total AFase gene signal intensity, respectively (Additional file 9). *Roseiflexus* sp. RS-1 belongs to the Chloroflexi phylum, and Roseiflexus species appeared to have the capacity for autotrophic CO_2_ fixation via the 3-hydroxypropionate pathway [2].

Among 42 individual vanillate demethylase genes, 26 were shared by both CO_2_ conditions, and four genes significantly increased under eCO_2_ with three (148499659 from *Sphingomonas wittichii* RW1, 90415596 from marine γ-Proteobacterium HTCC2207 and 13661652 from *Comamonas* *testosterone*) at *p*<0.05 and one (152985552 for *Pseudomonas aeruginosa* PA7) at *p* < 0.1.0 level. The shared genes had 92.2% of the total vanA gene signal intensity, and nine and seven appeared to be unique to aCO_2_ and eCO_2_, respectively. Those unique variants had 7.8% (4.1% for aCO_2_, and 3.7% for eCO_2_) of the total *vanA* gene signal intensity, and were detected in 3-6 of 12 samples at aCO2, and 3-5 of 12 at eCO_2_ (Additional file 10). There were three probes targeting three genes (110831199, 111611199, and 121552986) in *Verminephrobacter eiseniae* EF01-2 with the highest signal of 110831199 of 22.3% of total *vanA* gene signal intensity (a combined signal >25%). *V. eiseniae* was also detected as the most abundant population for the Pcc gene. Within the fourteen probes targeting *vanA* gene variants in *Sphingomonas wittichii* RW1, gene 118758991 and 148499659 ranked as the second and third most abundant *vanA* gene detected with 14.8% and 12.2% of total *vanA* gene signal intensity, and a significant (*p*<0.05) increase at eCO_2_ was observed in the gene 148499659 (a combined signal of those probes > 39.1%). *S. wittichii RW1* was isolated from the River Elbe, Germany, for its ability to completely mineralize the organic backbone of toxic dioxin pollutants, and to co-oxidize a large number of chlorinated congeners of both dibenzo- p -dioxin and dibenzofuran (Wittich et al., 1992 AEM). *Sphingomonas* species are found in a variety of habitats including soil, sediment, oceans, and lakes where they have a profound role in the degradation of large, complex aromatic compounds associated with decaying plant matter and chemical pollution (Halden et al., 1999 AEM). A significant increase at *p*<0.10 level was observed in the forth most abundant probe targets 152985552 (*Pseudomonas aeruginosa* PA7) with 9.3% of the total *vanA* gene signal. *Pseudomonas* species are common inhabitants of soil and water and can also be found on the surfaces of plants and animals. *P. aeruginosa* PA7 is an important soil bacterium with a complex metabolism capable of degrading polycyclic aromatic hydrocarbons, and producing interesting, biologically active secondary metabolites. The other two significant (*p*<0.05) increase genes were 90415596 from marine gamma proteobacterium HTCC2207 and 13661652 from *Comamonas testosterone* with 3.5% and 2.4% of the total *vanA* gene signal, respectively. Marine gamma proteobacterium HTCC2207 is a member of the SAR92 clade from Coastal Pacific Ocean, Newport, Oregon and *Comamonas testosteroni* is able to utilize a number of aromatic compounds as the sole carbon and energy sources. The populations of *S. wittichii*, *P. aeruginosa* and *C. testosteroni* significantly increased at eCO_2_, which is consistent with a significant (*p*<0.05) increase in *vanA* gene abundance at eCO_2_. This indicates that the utilization of lignin related aromatic compounds by those microorganisms in soil may increase at eCO_2_. However, the abundance of all of genes directly involved in lignin decomposition did not change significantly at eCO_2_, suggesting that recalcitrant carbon degradation may remain unaffected.

**2. Responses of nitrogen cycling genes to eCO_2_**

Based on the total signal intensity of 60 genes detected, *nirS* was found to be significantly (*p* < 0.05) increased at eCO_2_. Among 31 shared genes, two were significantly increased (74038408 and 81251659) at *p* < 0.05 level under eCO_2_. At the *p* < 0.1 level, two additional genes showed significant changes with one (77378647) increase and another (74038440) decrease at eCO_2_. Six and 23 *nirS* variants were only detected at aCO_2_ or eCO_2_, respectively (Additional file 1). Those variants had approximately 17.3% (4.1% for aCO_2_ and 13.2% for eCO_2_) of the total *nirS* signal intensity, and they were detected in 3-4 samples among 12 aCO_2_ samples, and 3-6 samples among 12 eCO_2_ samples (Additional file 1). Based on the number of unique genes detected and their signal intensities, those unique genes may play important roles in response to eCO_2_.

All top five *nirS* variants detected with about 4.27-7.82% of the total *nirS* gene signal intensity and all those significantly changed genes were derived from uncultured organisms. Only four detected genes were derived from cultured organisms including 68349021 (*Pseudomonas* sp. C10-2), 109454988 (*Roseobacter denitrificans* OCh 114), 87251639 (*Paracoccus sp.* R-27041), and 19548111 (*Thauera aromatica*). The first two variants were shared by aCO_2_ and eCO_2_ conditions but without significant changes at eCO_2_, and the last two were unique to aCO_2_ and eCO_2_, respectively (Additional file 11, Additional file 12). Based on the deduced amino acid sequences, some of the uncultured variants were closely related to known denitrifiers, such as *Paracoccus*, *Pseudomonas*, and *Thauera*, but most of them could not yet be defined.

**References:**

1. Aamand J, Ahl T, Spieck E: **Monoclonal antibodies recognizing nitrite oxidoreductase of *Nitrobacter hamburgensis*, *N*. *winogradskyi*, and *N*. *vulgaris***. *Appl Environ Microbiol* 1996, **62**(7):2352-2355.

2. Klatt CG, Bryant DA, Ward DM: **Comparative genomics provides evidence for the 3-hydroxypropionate autotrophic pathway in filamentous anoxygenic phototrophic bacteria and in hot spring microbial mats**. *Environmental Microbiology* 2007, **9**(8):2067-2078.

3. ROME S, FERNANDEZ MP, BRUNEL B, NORMAND P, CLEYET-MAREL J-C: ***Sinorhizobium medicae* sp. nov., Isolated from Annual Medicago spp**. *Int J Syst Bacteriol* 1996, **46**(4):972-980.

4. Kornacker MG, Boyd A, Pugsley AP, Plastow GS: ***Klebsiella pneumoniae* strain K21: evidence for the rapid secretion of an unacylated form of pullulanase**. *Molecular Microbiology* 1989, **3**(4):497-503.

5. Martinez D, Berka RM, Henrissat B, Saloheimo M, Arvas M, Baker SE, Chapman J, Chertkov O, Coutinho PM, Cullen D *et al*: **Genome sequencing and analysis of the biomass-degrading fungus *Trichoderma reesei* (syn. Hypocrea jecorina)**. *Nat Biotech* 2008, **26**(5):553-560.

6. Ward OP, Qin WM, Dhanjoon J, Ye J, Singh A: **Physiology and Biotechnology of *Aspergillus***. In: *Advances in Applied Microbiology.* Edited by Allen I. Laskin JWBGMG, Sima S, vol. Volume 58: Academic Press; 2005: 1-75.

7. Shallom D, Shoham Y: **Microbial hemicellulases**. *Current Opinion in Microbiology* 2003, **6**(3):219-228.
