# Supplementary material for: Elevated CO2 influences microbial carbon and nitrogen cycling
Source: BMC Microbiol. 2013 May 29;13:124. doi: 10.1186/1471-2180-13-124 (PMC3679978; doi:10.1186/1471-2180-13-124)
Supplement: Additional file 6 — A figure about the normalized signal intensities of glucoamylase encoding gene detected. [file 1471-2180-13-124-S6.doc]

*

*

150377998, *Sinorhizobium medicae* WSM41967157834, *Azotobacter vinelandii* AvOP12666724, *Talaromyces emersonii*87286596, *Blastopirellula marina* DSM 364514024312, *Mesorhizobium loti* MAFF30309991799470, *Nitrobacter hamburgensis* X1489949849, *Saccharophagus degradans* 2-40113729835, *Caulobacter* sp. K31126174562, *Shewanella baltica* OS1552788, *Amorphotheca resinae*

**Additional file 6** The normalized signal intensities of glucoamylase encoding gene detected. **P* < 0.10.
