# Supplementary material for: Elevated CO2 influences microbial carbon and nitrogen cycling
Source: BMC Microbiol. 2013 May 29;13:124. doi: 10.1186/1471-2180-13-124 (PMC3679978; doi:10.1186/1471-2180-13-124)
Supplement: Additional file 7 — A figure about the normalized signal intensities of pulA gene detected. [file 1471-2180-13-124-S7.doc]

**

*

68543224, *Shewanella baltica* OS15567535763, *Burkholderia vietnamiensis* G4111018069, *Rhodococcus* sp. RHA188794859, *Alteromonas macleodii* 'Deep ecotype'29605797, *Streptomyces avermitilis* MA-468015966601, *Sinorhizobium meliloti* 1021148838659, *Vibrio shilonii* AK1144898973, *Magnetospirillum gryphiswaldense* MSR-1145577320, *Pseudomonas mendocina* ymp116749778, *Syntrophobacter fumaroxidans* MPOB71366297, *Nocardioides* sp. JS61463256935, *Pseudomonas syringae* pv. *syringae* B728a84383828, *Janibacter* sp. HTCC264941407368, *Mycobacterium avium* subsp. *paratuberculosis* K-10126710975, *Sagittula stellata* E-3769284809, *Kineococcus radiotolerans* SRS3021668056074, *Exiguobacterium sibiricum* 255-15149182592, *Bacillus* sp. SG-115805432, *Deinococcus radiodurans* R189067885, *Oceanicola granulosus* HTCC2516157075933, *Streptococcus gordonii* str. Challis substr. CH139650229, *Rhodopseudomonas palustris* CGA00971366738, *Nocardioides* sp. JS61483643155, *Hahella chejuensis* KCTC 2396

**Additional file 7** The normalized signal intensities of *pulA* gene detected. ***P* < 0.05, **P* < 0.10.
