# Supplementary material for: Elevated CO2 influences microbial carbon and nitrogen cycling
Source: BMC Microbiol. 2013 May 29;13:124. doi: 10.1186/1471-2180-13-124 (PMC3679978; doi:10.1186/1471-2180-13-124)
Supplement: Additional file 8 — A figure about the normalized signal intensities of endoglucanase gene detected. [file 1471-2180-13-124-S8.doc]

*

55295400, *Irpex lacteus*31747164, *Hypocrea jecorina*66845524, *Aspergillus fumigatus* Af29377176916, *Hypocrea jecorina*4249556, *Humicola grisea* var. *thermoidea*144770, Uncultured bacterium144416, Uncultured bacterium58709678, *Phanerochaete chrysosporium*87309129, *Blastopirellula marina* DSM 3645

**Additional file 8** The normalized signal intensities of endoglucanase gene detected. **P* < 0.10.
