# Supplementary material for: Elevated CO2 influences microbial carbon and nitrogen cycling
Source: BMC Microbiol. 2013 May 29;13:124. doi: 10.1186/1471-2180-13-124 (PMC3679978; doi:10.1186/1471-2180-13-124)
Supplement: Additional file 9 — A figure about the normalized signal intensities of ara gene detected. [file 1471-2180-13-124-S9.doc]

**

**

*

*

113733431, *Caulobacter* sp. K31

106889690, *Roseiflexus* sp. RS-1

154297971, *Botryotinia fuckeliana* B05.10

83773727, *Aspergillus oryzae*

21220080, *Streptomyces coelicolor* A3(2)

156934116, *Enterobacter sakazakii* ATCC BAA-894

120577985, *Penicillium capsulatum*

85831189, *Leeuwenhoekiella blandensis* MED217

29610420, *Streptomyces avermitilis* MA-4680

15212230, *Cellvibrio japonicas*

153890549, *Opitutaceae bacterium* TAV2

46205560, *Magnetospirillum magnetotacticum* MS-1

121709492, *Aspergillus clavatus* NRRL 1

1244586, *Aspergillus niger*

67985747, *Kineococcus radiotolerans* SRS30216

23465129, *Bifidobacterium longum* NCC2705

116623106, *Solibacter usitatus* Ellin6076

16767843, *Geobacillus stearothermophilus*

29340972, *Bacteroides thetaiotaomicron* VPI-5482

150385878, *Victivallis vadensis* ATCC BAA-548

116208630, *Chaetomium globosum* CBS 148.51

116621213, *Solibacter usitatus* Ellin6076

86196158, *Magnaporthe grisea* 70-15

39956518, *Magnaporthe grisea* 70-15

1054936, *Trichoderma koningii*

89044975, *Oceanicola granulosus* HTCC2516

40557184, *Aureobasidium pullulans*

156868874, *Clostridium leptum* DSM 753

94967359, *Acidobacteria bacterium* Ellin345

149936622, *Parabacteroides distasonis* ATCC 8503

17936815, *Agrobacterium tumefaciens* str. C58

115385653, *Aspergillus terreus* NIH2624

89047094, *Oceanicola granulosus* HTCC2516

66574689, *Xanthomonas campestris* pv. *campestris* str. 8004

28919715, *Neurospora crassa* OR74A

116608903, *Arthrobacter* sp. FB24

**Additional file 9** The normalized signal intensities of *ara* gene detected. ***P* < 0.05, **P* < 0.10.
