# Supplementary material for: Elevated CO2 influences microbial carbon and nitrogen cycling
Source: BMC Microbiol. 2013 May 29;13:124. doi: 10.1186/1471-2180-13-124 (PMC3679978; doi:10.1186/1471-2180-13-124)
Supplement: Additional file 10 — A figure about the normalized signal intensities of vanA gene detected. [file 1471-2180-13-124-S10.doc]

**

*

**

**

110831199, *Verminephrobacter eiseniae* EF01-2

148499659, *Sphingomonas wittichii* RW1

118758991, *Sphingomonas wittichii* RW1

152985552, *Pseudomonas aeruginosa* PA7

90415596, marine gamma proteobacterium HTCC2207

148501120, *Sphingomonas wittichii* RW1

13661652, *Comamonas testosterone*

118701991, *Sphingomonas wittichii* RW1

49529682, *Acinetobacter* sp. ADP1

114050057, *Streptomyces ambofaciens*

109455731, *Roseobacter denitrificans* OCh 114

111611199, *Verminephrobacter eiseniae* EF01-2

27350645, *Bradyrhizobium japonicum* USDA 110

78519228, *Bradyrhizobium* sp. BTAi1

118762970, *Sphingomonas wittichii* RW1

66572050, *Xanthomonas campestris* pv. *campestris* str. 8004

111147441, *Frankia alni* ACN14a

118762773, *Sphingomonas wittichii* RW1

113733254, *Caulobacter* sp. K31

148554462, *Sphingomonas wittichii* RW1

28853329, *Pseudomonas syringae* pv. *tomato* str. DC3000

88865992, *Jannaschia* sp. CCS1

91781025, *Burkholderia xenovorans* LB400

91795559, *Chromohalobacter salexigens* DSM 3043

91795567, *Chromohalobacter salexigens* DSM 3043

3334811, *Streptomyces coelicolor* A3(2)

1790867, *Comamonas testosterone*

148501358, *Sphingomonas wittichii* RW1

152995325, *Marinomonas* sp. MWYL1

148553989, *Sphingomonas wittichii* RW1

148499796, *Sphingomonas wittichii* RW1

121552986, *Verminephrobacter eiseniae* EF01-2

23492471, *Corynebacterium efficiens* YS-314

148556298, *Sphingomonas wittichii* RW1

148501505, *Sphingomonas wittichii* RW1

118758093, *Sphingomonas wittichii* RW1

118762756, *Sphingomonas wittichii* RW1

71735530, *Pseudomonas syringae* pv. *phaseolicola* 1448A

91786893, *Polaromonas* sp. JS666

149814105, *Plesiocystis pacifica* SIR-1

87199889, *Novosphingobium aromaticivorans* DSM 12444

91686245, *Burkholderia xenovorans* LB400

**Additional file 10** The normalized signal intensities of *vanA* gene detected. ***P* < 0.05, **P* < 0.10.
