# Supplementary material for: Elevated CO2 influences microbial carbon and nitrogen cycling
Source: BMC Microbiol. 2013 May 29;13:124. doi: 10.1186/1471-2180-13-124 (PMC3679978; doi:10.1186/1471-2180-13-124)
Supplement: Additional file 11 — A figure about the normalized signal intensities of shared nirS gene detected. [file 1471-2180-13-124-S11.doc]

**

**

*

*

57335474, uncultured bacterium

76577396, uncultured bacterium

68349021, *Pseudomonas* sp. C10-2

46850218, uncultured bacterium

68164720, uncultured bacterium

74038370, uncultured bacterium

87281376, uncultured bacterium

28542625, uncultured bacterium

77378459, uncultured bacterium

77378443, uncultured bacterium

74038408, uncultured bacterium

32895106, uncultured bacterium

81251659, uncultured organism

74038388, uncultured bacterium

77378719, uncultured bacterium

77378465, uncultured bacterium

7160891, uncultured bacterium wC1

77378647, uncultured bacterium

83318864, uncultured bacterium

62084130, uncultured bacterium

28542601, uncultured bacterium

81251655, uncultured organism

77378779, uncultured bacterium

77820032, uncultured organism

74038306, uncultured bacterium

28542633, uncultured bacterium

77820052, uncultured organism

109454988, *Roseobacter denitrificans* OCh 114

77378749, uncultured bacterium

76577390, uncultured bacterium

74038440, uncultured bacterium

**Additional file 11** The normalized signal intensities of shared *nirS* gene detected. ***P* < 0.05, **P* < 0.10.
