# Supplementary material for: Elevated CO2 influences microbial carbon and nitrogen cycling
Source: BMC Microbiol. 2013 May 29;13:124. doi: 10.1186/1471-2180-13-124 (PMC3679978; doi:10.1186/1471-2180-13-124)
Supplement: Additional file 12 — A table listing the nirS genes only detected at aCO2 or eCO2. [file 1471-2180-13-124-S12.doc]

**Additional file 12** The *nirS* genes only detected at aCO_2_ or eCO_2_.

|  | Genbank ID | Organism | No. of sample*^a^* | Abundance*^b^* |
| --- | --- | --- | --- | --- |
| aCO_2_ | 68510354 | uncultured bacterium | 3 | 3.45 |
|  | 51534821 | uncultured bacterium | 3 | 2.38 |
|  | 77378451 | uncultured bacterium | 3 | 0.90 |
|  | 46850308 | uncultured bacterium | 3 | 1.52 |
|  | 7160889 | uncultured bacterium wB32 | 3 | 7.00 |
|  | 87251639 | *Paracoccus* sp. R-27041 | 4 | 2.61 |
| eCO_2_ | 77378641 | uncultured bacterium | 3 | 1.29 |
|  | 50838773 | uncultured bacterium | 3 | 1.43 |
|  | 28542575 | uncultured bacterium | 3 | 1.148 |
|  | 74038296 | uncultured bacterium | 3 | 1.23 |
|  | 32895210 | uncultured bacterium | 3 | 1.39 |
|  | 77378791 | uncultured bacterium | 3 | 1.11 |
|  | 74038278 | uncultured bacterium | 3 | 3.10 |
|  | 76577432 | uncultured bacterium | 3 | 0.93 |
|  | 116013290 | uncultured bacterium | 3 | 1.96 |
|  | 116013326 | uncultured bacterium | 3 | 1.53 |
|  | 74038368 | uncultured bacterium | 3 | 1.23 |
|  | 77378681 | uncultured bacterium | 3 | 1.54 |
|  | 77378473 | uncultured bacterium | 3 | 0.95 |
|  | 68510310 | uncultured bacterium | 4 | 1.97 |
|  | 28542655 | uncultured bacterium | 4 | 7.82 |
|  | 74038380 | uncultured bacterium | 4 | 1.81 |
|  | 77378747 | uncultured bacterium | 4 | 2.39 |
|  | 87281179 | uncultured bacterium | 5 | 1.94 |
|  | 68510348 | uncultured bacterium | 5 | 2.53 |
|  | 77378731 | uncultured bacterium | 5 | 5.83 |
|  | 32895084 | uncultured bacterium | 5 | 3.69 |
|  | 57335576 | uncultured bacterium | 6 | 8.54 |
|  | 19548111 | *Thauera aromatica* | 4 | 1.85 |

*a*. The number of samples detected out of 12; *b*. The total normalized signal intensity.
