# Supplementary material for: Elevated CO2 influences microbial carbon and nitrogen cycling
Source: BMC Microbiol. 2013 May 29;13:124. doi: 10.1186/1471-2180-13-124 (PMC3679978; doi:10.1186/1471-2180-13-124)
Supplement: Additional file 13 — The supplemental descriptions for materials and methods. [file 1471-2180-13-124-S13.docx]

**Additional file 13. Materials and Methods**

**1. Site, sampling and environmental variable analyses**

This study was conducted within the BioCON experiment site [1] located at the Cedar Creek Ecosystem Science Reserve, MN, USA, in which three main treatments: CO_2_ (ambient, 368 µmol^-1^ vs elevated, 560 µmol^-1^), N (ambient vs 4 g m^-2^ supply per year), and plant diversity (1, 4, 9 and 16 species) were performed. In this study, soil samples from 24 plots (12 biological replicates from ambient CO_2_ and 12 biological replicates from elevated CO_2_. All with 16-species and no additional N supply) were collected in July 2007. Each sample was composted from five bulk soil cores at a depth of 0-15 cm and transported to the laboratory immediately, frozen and stored at -80^o^C until DNA extraction for GeoChip analysis. The aboveground and belowground biomass, plant carbon and nitrogen concentrations, soil pH, moisture, total soil carbon and nitrogen concentrations, and *in situ* net nitrogen mineralization and net nitrification were measured as previously described [1-2].

**2. GeoChip analysis**

Soil DNA was extracted by freeze-grinding mechanical lysis as described previously [3], and was purified using a low melting agarose gel purification method followed by phenol extraction. DNA amplification and labeling, as well as the purification of labeled DNA, were carried out according the methods described by [4]. GeoChip 3.0 was used to analyze the functional structure of the soil microbial communities. Before hybridization, the labeled and purified DNA was suspended in hybridization solution contained 50% formamide, 3×SSC, 0.3% SDS, 0.7 μg of unlabeled herring sperm DNA and 0.85 mM dithiothreitol (DTT). This solution was heated at 98 °C for 3 min and then kept at 65 °C until the hybridization started. All hybridizations were carried out in triplicate at 42 °C for 10 hours. In this process, an additional denaturation at 95 °C for 1 min with agitation was performed after a 45-min prehybridization. In past hybridization washes processes, one-time wash at 23 °C for 20 sec with wash buffer II (0.1×SSC, 0.1% SDS) was added before three-time washes at 23 °C for 10 sec with wash buffer III (0.1×SSC). After washing and drying, the arrays were scanned by ScanArray Express Microarray Scanner (Perkin Elmer, Boston, MA) at 633 nm using a laser power of 90% and a photomultiplier tube (PMT) gain of 75%. ImaGene version 6.0 (Biodiscovery, El Segundo, CA) was then used to determine fluorescence image intensity and background intensity, as well as to identify spots of poor quality. Raw data from ImaGene were submitted to Microarray Data Manager in our website (<http://ieg.ou.edu/microarray/>) and analyzed using data analysis pipeline with the following major steps: (i) The spots flagged as 1 or 3 by Imagene and with a signal to noise ratio (SNR) less than 2.0 were removed as poor- quality spots. After bad spot’s cleaning, normalized intensity of each spot was calculated by dividing the signal intensity of each spot by the mean intensity of the sample. For outlier removal, if any of replicates had (signal–mean) more than two times the standard deviation, this replicate was moved. This process continued until no such replicates were identified. Finally, at least 0.34 time of the final positive spot (probe) number (minimum of two spots) was required for a particular gene.

**3. Statistical analysis**

The matrices of microarray data resulting from our pipeline were considered as ‘species’ abundance in statistical analyses. The dataset containing the functional genes shared by at least three samples in the biological replicate was applied to do further analysis. Direct and indirect multivariate ordination analyses were carried using PC-ORD for Windows [5] and confirmed by CANOCO 4.5 for Windows (Biometris – Plant Research International, The Netherlands). Detrended correspondence analysis (DCA) [6] , combined with analysis of similarities (ANOSIM), non-parametric multivariate analysis of variance (Adonis) and Multi-Response Permutation Procedure (MRPP), was used to determine the overall functional changes in the microbial communities. To eliminate the potential bias caused by filling, normalized intensities with filling small values or binary data for gene present or absent were used to do the statistical analyses. The effects of elevated CO_2_ on functional microbial communities, microbial processes, and environmental parameters were analyzed by computing the response ratio (RR) using the formula described by Luo et al. [7]. The total abundance of each gene category or family was simply the sum of the normalized intensity for the gene category or family. For comparing the relative abundances of different organisms, the total abundance values were calculated by summing the normalized intensity of the genes detected for this organism.

Redundancy analysis (RDA) was carried to reveal the individual or a set of environmental variables significantly explained the variation in functional microbial communities using CANOCO 4.5 for Windows (Biometris – Plant Research International, The Netherlands). Meanwhile, RDA with forward selection and unrestricted Monte Carlo permutation test based on 999 random permutations of the residuals under the full regression model were used to select the minimum number of environmental variables explaining the largest amount of variation in the model. The relative contribution of individual environmental variables to the ordination axes was evaluated by canonical coefficients (significance of approximate *t*-tests) and intraset correlations. Unrestricted Monte Carlo permutation tests (999 permutations, *p*≤0.05) were used to test the statistical significance of the first 2 ordination axes. In order to evaluate the specific contribution of each significant variable, a variation partitioning analysis was run with the variables of interest as explanatory variables and the other significant variables as covariables [8-9]. Correlations between the matrixes were calculated and tested for significance (*p*-value) using Mantel test in PC-ORD for Windows. Significant Pearson’s linear correlation (*r*) analysis, and analyses of variance (ANOVA) were carried out in SPSS 16.0 for windows (SPSS Inc., Illinois, USA).

**References:**

1. Reich PB, Knops J, Tilman D, Craine J, Ellsworth D, Tjoelker M, Lee T, Wedin D, Naeem S, Bahauddin D *et al*: **Plant diversity enhances ecosystem responses to elevated CO_2_ and nitrogen deposition**. *Nature* 2001, **410**(6830):809-812.

2. Reich PB, Hobbie SE, Lee T, Ellsworth DS, West JB, Tilman D, Knops JMH, Naeem S, Trost J: **Nitrogen limitation constrains sustainability of ecosystem response to CO_2_**. *Nature* 2006, **440**(7086):922-925.

3. Zhou J, Bruns MA, Tiedje JM: **DNA recovery from soils of diverse composition**. *Appl Environ Microbiol* 1996, **62**(2):316-322.

4. Xu M, Wu WM, Wu L, He Z, Van Nostrand JD, Deng Y, Luo J, Carley J, Ginder-Vogel M, Gentry TJ *et al*: **Responses of microbial community functional structures to pilot-scale uranium in situ bioremediation**. *ISME J* 2010, **4**(8):1060-1070.

5. McCune B, Mefford MJ: **PC-ORD: multivariate analysis of ecological data. Version 4. User’s guide. MjM Software Design, Gleneden Beach, Oregon.** 1999.

6. Hill MO, Gauch HG: **Deterended correspondence analysis, an improved ordination technique**. *Vegetatio* 1980, **42**(47-58).

7. Luo Y, Hui D, Zhang D: **Elevated CO_2_ stimulates net accumulations of carbon and nitrogen in land ecosystems: a meta-analysis**. *Ecology* 2006, **87**(1):53-63.

8. Ramette A: **Multivariate analyses in microbial ecology**. *Fems Microbiology Ecology* 2007, **62**(2):142-160.

9. Ramette A, Tiedje JM: **Multiscale responses of microbial life to spatial distance and environmental heterogeneity in a patchy ecosystem**. *Proceedings of the National Academy of Sciences* 2007, **104**(8):2761-2766.
